# Experiences of undergoing cardiac surgery among older people diagnosed with postoperative delirium: one year follow-up

**DOI:** 10.1186/s12912-015-0069-7

**Published:** 2015-03-30

**Authors:** Helena Claesson Lingehall, Nina Smulter, Birgitta Olofsson, Elisabeth Lindahl

**Affiliations:** 1 Department of Nursing, Umeå University, Umeå, SE-901 87 Sweden; 2 Department of Surgical and Perioperative Science, Cardiothoracic Surgery Division, Umeå, SE-901 85 Sweden

**Keywords:** Cardiac surgery, Delirium, Lived experience, Nursing, Patient’s experience, Perioperative period, Older people

## Abstract

**Background:**

Cardiovascular disease is common among old people and many undergo cardiac surgery. Scientific knowledge is available on cardiac surgery from several perspectives. However, we found few studies focusing on older patients’ experiences of cardiac surgery. The aim of this study was to illuminate experiences of undergoing cardiac surgery among older people diagnosed with postoperative delirium, a one year follow-up.

**Methods:**

Qualitative interviews were conducted with 49 participants (aged ≥70 years) diagnosed with delirium after cardiac surgery. Data were collected in Sweden during 2010 through individual, semi-structured interviews in participants’ homes one year after surgery. The interviews were analyzed using qualitative content analysis.

**Results:**

Four themes with sub-themes were formulated: *Feeling drained of viability* includes having a body under attack, losing strength and being close to death. *Feeling trapped in a weird world* describes participants having hallucinations, being in a nightmare and being remorseful for their behavior. *Being met with disrespect* includes feeling disappointed, being forced, and feeling like cargo. On the other hand, *Feeling safe*, including being in supportive hands and feeling grateful, points to participants’ experiences of good care and the gift of getting a second chance in life.

**Conclusions:**

Even one year after cardiac surgery, participants described in detail feelings of extreme vulnerability and frailty. They also had felt completely in the hands of the health care professionals. Participants described experiences of hallucinations and nightmares during hospitalization. Cardiac surgery was a unique, fearful, traumatic and unpleasant experience yet could also include pleasant or rewarding aspects. It seems that health care professionals need deeper knowledge on postoperative delirium in order to prevent, detect and treat delirium to avoid and relieve the suffering these experiences might cause.

## Background

Cardiac surgery can save lives, but can also mean increased risks of unexpected mental and physical complications, both during surgery and after discharge [1-3]. The risk of being affected by serious complications or even the risk of dying after cardiac surgery is gradually declining due to the fact that patients accepted for surgery are steadily becoming older [4]. Some studies have described patients’ experiences of cardiac surgery as a major life- threatening event. These events come with a strong encroachment on the person’s body and integrity [5-7].

Neuropsychiatric symptoms, such as acute confusional states (delirium), mostly occur close to surgical procedures, in general, as a period of various feelings of discomfort and distress [8]. Delirium is not a disease, but a neuropsychiatric syndrome. Four key features characterize delirium: a disturbance of consciousness; a change in cognition; an acute onset, within hours or days; and fluctuations during the day [9]. Since the early 1960s, delirium has been described as a common problem after cardiac surgery with cardiopulmonary bypass (CPB) [10]. The incidence of delirium related to cardiac surgery has been shown to be 43–54% [11,12]. Old age is one paramount risk factor for developing delirium, together with a number of risk factors such as diabetes, volume load during operation [13] and prolonged mechanical ventilation [14]. Studies have shown that delirium also can be associated with more hospital readmissions, increased mortality [15], long-term cognitive changes [16,17] and functional decline [11,18]. A multi-component intervention program managed by an interdisciplinary team can prevent delirium in hospitalized older patients [19,20].

Health care professionals have extensive knowledge about treating cardiovascular disease and performing cardiac surgery procedures. However, older patients are more fragile and vulnerable with multiple morbidities and, hence, place greater demands on health care professionals’ skills and knowledge. This may influence older patients’ perioperative outcomes [21]. Studies have shown that nurses’ knowledge of delirium is inadequate [22,23]. Moreover, nurses who care for patients with delirium are unable to understand and adequately respond to patients’ behavior and statements, because they cannot understand patients’ feelings and discomfort [8]. Likewise, nurses have described discomfort when talking about caring for delirious patients, and mentioned their own experiences of frustration, irritation, guilt and anxiety [24].

Delirium is described in other settings from several perspectives, but few studies describe experiences of cardiac surgery among older patients diagnosed with postoperative delirium. To our knowledge, no previous study specifically focused on older peoples’ experiences one year after surgery. Therefore, the aim was to illuminate experiences of undergoing cardiac surgery among older people diagnosed with postoperative delirium, a one year follow-up.

## Methods

### Participants and setting

The sample comprised 17 women and 32 men, a total of 49.The age of participants ranged from 71 to 91 years (median 78 years). They were all living independently, and all could speak and understand Swedish. Participants were recruited from a cohort study comprising patients who had undergone cardiac surgery (coronary bypass grafting, valve replacement or a combination thereof, n = 142) [12]. Inclusion criterion in the present study was: a diagnosis of postoperative delirium according to the Diagnostic and Statistical Manual of Mental Disorders IV- TR (DSM-IV-TR) [9].

### Data collection

An interview guide was constructed containing five semi-structured questions. Individual interviews were conducted in participants’ homes by two of the authors during 2010, one year after surgery (HCL, NS). The opening question was “Please, tell me how you experienced your hospitalization”? Further, follow-up questions were asked to encourage participants to share their experiences and facilitate talking about them. Questions included preoperative information, memories of the first postoperative days and memories of being confused, thoughts and feelings the participants did not usually have, and whether they had had any experiences of dreams or visions. The interviews lasted between 9 and 32 minutes (mean = 18 minutes).

### Analysis

The authors used qualitative content analysis inspired by Graneheim and Lundman [25]. Qualitative content analysis is a systematic way to describe content in verbal or written communication, giving emphasis to differences and similarities in the text [25,26].

Interviews were digitally recorded and transcribed verbatim. Analyses were performed in several steps. First, all texts were read through to get a sense of the whole. The text was then divided into meaning units, words or sentences related to each other through their content and context. Meaning units were condensed without losing the core meaning, and labelled with a code. Codes were sorted into eleven sub-themes, and then abstracted to four themes, as the richness of data made it possible to formulate latent meaning at an interpretative level. Qualitative content analysis involves going back and forth between meaning units, codes, sub-themes and themes. All authors discussed every step in the analysis process until consensus was achieved.

### Ethical considerations

This study was conducted according to the ethical principles guideline described in the Helsinki Declaration [27]. Ethics approval was given by Regional Ethical Review Board in Umeå, Sweden (Dnr 08-169 M, Dnr 2010-34-32). Participants received written and verbal information about the aim of the study two weeks before the interviews. Participation was voluntary. They were informed they could withdraw from the study at any time without giving an explanation, and they were assured of confidentiality. Participants were also informed that the two interviewers had been involved in their previous care during cardiac surgery one year earlier. Great attention was paid during the interviews to participants’ reactions as there is always a risk that some questions could be invasive.

## Results

Participants’ experiences of cardiac surgery were formulated as the four themes: *Feeling drained of viability*; *Feeling trapped in a weird world*; *Being met with disrespect*; and *Feeling safe*. Each theme had several sub-themes. Themes and sub-themes are presented in Table 1 and illuminated by quotations from the interview texts.

**Table 1:** **Themes and sub-themes**

Themes	Sub-themes
Feeling drained of viability	Having a body under attack
	Losing strength
	Being close to death
Feeling trapped in a weird world	Having hallucinations
	Being in a nightmare
	Being remorseful
Being met with disrespect	Feeling disappointed
	Being forced
	Feeling like cargo
Feeling safe	Being in supportive hands
	Feeling grateful

### Feeling drained of viability

Participants described how cardiac surgery was exhausting, terrifying and distressful. The surgery had made them aware of how fragile they were and how much they depended on help from healthcare professionals. Recovering from cardiac surgery became a big challenge. Lacking the power to manage daily activities became a negative experience. Sub-themes related to the theme *Feeling drained of viability* were: ‘having a body under attack’, ‘losing strength’, and ‘being close to death’.

#### Having a body under attack

Participants described how they felt their bodies were under attack. Their whole bodies were marked by illness and memories and signs of surgical procedures. For example, they experienced being tormented with needles everywhere and they could do nothing to prevent it. They described how they had experienced being defenseless when being suddenly assailed by illness. They thought they had had no choice but to put up with what was going on now, that their bodies were “useless”. Participants described bodily reactions such as visual changes, speaking difficulties, swollen legs, unsteady legs, fainting and constipation. They had even wished to die when their bodily attacks were severe.

“I fervently hoped I would die. I think it was unfair of God not to let it happen. I couldn’t even eat”. (female, 73)

Other bodily reactions experienced were thirst and loss of appetite, both described as awful and terrible. The smell of food induced nausea and vomiting. As eating was important for recovering, participants described it as a relief when their appetite returned.

Participants perceived their inability to meet their basic needs as a failure, for example their difficulty to breathe or failing to urinate. Some participants had cardiac arrest or fibrillation and others caught infections. When their body would not stop bleeding, more surgery was needed. These were described as terrifying experiences.

“…I couldn’t get any air. I couldn’t make a sound. I couldn’t breathe. I thought to myself, now I know what it feels like to be a perch stranded on dry land”. (male, 71)

Participants described how pain racked the body, sometimes with full strength. Other times, the pain was not as strong as expected. When medication was able to diminish the pain or even take it away, this was experienced as relief. Even with potent analgesics, the pain might not go away, leaving some participants dazed and disconnected from reality, with only fragmented memories of cardiac surgery. They felt out of control and later had to ask relatives, fellow patients or staff what had happened. When fellow patients had pain or other bodily discomfort, this was tough also for the participants.

“I arrived on Sunday evening, was given a needle on Monday evening, and something else. Then I can’t remember anything that happened for 2 weeks after”. (female, 77)

#### Losing strength

Participants described how their entire bodies were drained of strength and how they felt their bodies in a way collapsed after surgery. It was a frustrating and bothersome time. When their body felt exhausted, weak, dizzy and fatigued, they hardly managed anything but to keep their eyes open. Being unable to manage their daily hygiene, the participants felt fragile, handicapped and hopeless. The zest of life was gone. They did not even have strength to return to bed on their own after being in the bathroom; a nurse had to be of assistance.

Participants described their experience when strength was gone, and their fatigue was pronounced. They felt totally in the hands of the staff, became dependent on care and needed to remain in the hospital. Participants were accustomed to taking care of activities of daily living and when they could not care for their basic needs, they became embarrassed and felt they were a burden. They said they did not want to be a ‘bother’. And, they felt that they were sabotaging the goodwill of staff and overloading their workday.

“….I felt ashamed that I had to pee so often. I apologized for calling them, but they said, ‘You needn’t apologize; we’re here to help you’, and ‘We’ll come and help you however many times you need us’, but it felt so unpleasant”. (female, 81)

#### Being close to death

Participants described how their bodies were extremely vulnerable and how their life had become fragile. They realized they might become paralyzed, end up in a “vegetative state” or even never wake up again. This was a terrifying thought. After surgery, it was also distressful to realize that the procedure had not been easy, that the surgery had been more complicated than expected. This made the participants realize that they actually had been close to death.

“….my heart stopped, but they got it going again. They said that sometimes that happens, so I was happy when I woke up. It certainly didn’t feel nice. I wasn’t afraid, no…… But you start thinking, ‘What if I had died?” (female, 73)

### Feeling trapped in a weird world

Several participants described how they, mostly in the evening and at night during their post- operative hospitalization, suddenly felt immersed in a strange world. They described it as something unpleasant and terrifying, but it could also be a pleasant experience. They felt dragged into a strange world of dreams, such as they had never experienced before. Some participants described themselves as dreamy, woozy and confused. Others said they felt completely clear and lucid though they “were” in a strange world. Participants also described how their own manners could change into something unpleasant, bothersome and embarrassing. These were painful memories which, according to their descriptions, still bothered them one year later.

Sub-themes related to the theme *Feeling trapped in a weird world* were: ‘having hallucinations’, ‘being in a nightmare’ and ‘being remorseful’.

#### Having hallucinations

Participants described how they saw and imagined strange things and found them quite nice, funny or, at worst, slightly odd. They talked about a range of delusions such as police investigations, yellow wagons rolling, bus garages or railway stations, or geometric patterns, colorful and intense, which they saw on floors, walls and ceilings. One participant described how deceased relatives had shouted and beckoned her over when she felt she had returned from the other side, from death. However, it was quite a nice memory. Encountering small, helpful and nice people such as gnomes or seeing dead relatives was described as a pleasant and consoling experience.

“I think I was clear and everything; I had firm opinions. But I was in a dream world actually, because I was at another hospital the whole time. They could never get rid of me. Even now, I know I was at the other hospital”. (male, 83)

#### Being in a nightmare

Participants described how their hospitalization could change into a nightmare with strong emotional feelings of frustration, anger, pronounced fear, anxiety and loneliness. Ordinary things could suddenly appear dangerous and unsafe without any reason, e.g. staff and fellow patients became silhouettes. Meeting new people suddenly became upsetting. Participants described how they continued being misunderstood and failed to get the staff’s attention during the nightmares. They constantly had to ask members of staff where they were or what time it was. By “nightmares” they meant a range of life-threatening experiences such as being chased and threatened with their lives, being trapped in a cage, crying or trying to cry for help and not being heard or seen, watching a blood pressure cuff turn into a bomb, or seeing family members being hurt. Various threatening animals appeared in the nightmare, turning it into a horror-filled experience. The participant below described his nightmares like a horror movie that went on for days, which is similar to other participants’ experiences.

“He [the male nurse] was really great… So I managed to give myself the idea that he was out to crush me. Then I got nightmares about how I was in Hell. It was glowing… and they were crawling all over me – like, you know, some kind of cockroaches – and they just kept on multiplying, and hordes and hordes of them swarmed all over me. It was awful! I thought it was real … the dream was, when I was wheeled out of the room: I was supposed to go for some radiation – how they lied to me, saying there was nothing wrong with me. I saw the end coming closer and closer. I understood they were whisking me away to a burial, my own burial, and how I then, like … they whisked me away, since I was becoming more and more alone. Finally I was completely gone… There were so many episodes like that, that I dreamed and somehow fantasized about – I was in another world. I really thought I was losing my mind”. (male, 75)

Participants said that they became depressed, mentally affected and thought they were going totally insane for real. It was a relief and liberation when the nightmares stopped. Participants became confident in the weird world when it lasted for several days or when they realized that they were not alone. Some participants still felt trapped and described anxiety and worries over their weird experiences one year after surgery as no-one had explained their condition and the common symptom of delirium to them. When their fellow patients went through a similar experience of being stuck in a weird world, they experienced this as tough and unpleasant too. There were participants who stated that they were confused but who were unable to describe it. However, there were also participants who did not experience anything unusual or weird during cardiac surgery.

#### Being remorseful

Participants talked about having to apologize for their own behaviour and described this as a difficult and shameful thing to do. Often, it was strange behaviour they were not even properly aware of, which they exhibited during times when they felt their body was under attack or while they were trapped in a weird world. They also expressed shame when they could not remember which staff member they had offended, and hence could not apologize. Participants felt really awful when relatives told them that they were ashamed of their behaviour. When they had felt trapped in a weird world, they had felt restless and had sometimes reacted in an unruly way. Some had not followed the treatment recommendations and, as a consequence, had to undergo additional operations. They felt remorseful over having negatively impacted surgery outcomes, especially because they felt the staff had worked so hard to help them.

“They say I was ranting and raving a bit there. I don’t remember anything of that. But I do know one thing. I hit one of the nurses right on the chest, like this. I remembered that when she came into the room….. and I asked her if she was the one I’d hit. She said ‘Yes’, and I apologized. I felt horrible about it….. When I think about it I feel awful [starts to cry]. I’ve thought about this a few times, how I could possibly do such a thing [pauses]. But it’s over and forgotten, I hope”. (male, 74)

There were also participants who were afraid and ashamed of their strange behaviour and did not dare ask for an explanation, and, hence, kept it as a secret.

### Being met with disrespect

Participants expressed how cardiac surgery was frustrating, unpleasant and bothersome. It sometimes made them question their confidence in health professionals and health care. Sub-themes related to the theme *Being met with disrespect* were: ‘feeling disappointed’, ‘being forced’ and ‘feeling like cargo’.

#### Feeling disappointed

Participants described how they had not always felt well looked after and had not always received the good professional care they were expecting. When recovery from surgery became a greater challenge than expected, they became disappointed. They described how they were not always given the right treatment, how they had fallen on the floor or out of their bed, sometimes resulting in injuries and prolonged hospitalization. One participant expressed disappointment over being deceived, when she was told that the operation was easy even though it was not.

“…I was so angry. I was enraged. I was disappointed that I was feeling so horrible. I couldn’t stand the fact that I felt so awful. No doubt that was what manifested itself there, as I thought I was feeling too sick after the operation compared with what I had been before – because if I hadn’t had the operation, I would have managed fine, I thought”. (female, 77)

One participant described how a nurse had made ridiculed of him when he asked questions about his weird experiences. He said this disappointed him as he thought it disrespectful. Participants expressed disappointment about not getting appropriate information. They felt they should have been told about the risk of complications. They should have been warned before the surgery that they might suffer delirium. Also, not having the chance to meet the surgeon and thank him or her for the surgery was disappointing.

“…that I was never introduced to the surgeon who performed the operation on me or to the physicians. No-one said anything, so every time I saw a physician standing there looking at me I wondered if was him”. (female, 72)

The hospital was described as a suboptimal environment for rest and recovery. Sleeping was difficult when the room was cold. A participant mentioned a broken toilet and the anxiety this caused him. Several participants said it was a strange and disturbing experience to have male and female patients sleeping in the same room.

“…one of them wants to have everything bright and the other wants it to be dark, while the third is restless at night”. (male, 90)

#### Being forced

Participants described how they felt they were forced to walk, even though they said they could not, forced to eat when they had lost their appetite or felt nauseous, and told to sleep when they could not sleep.

“My first time in the chair, they tried to get me to walk, so I said right out, ‘I can’t do it’. So they told me to aim for the wall, stand up and walk toward the walking chair. And I toppled over, of course – I knew my balance was no good. Then suddenly, bang!, there I was, sprawled under the next bed”. (male, 74)

One participant’s only memory of cardiac surgery was that the nurses were determined to make him listen and he felt tortured when they told him to calm down.

#### Feeling like cargo

Participants described how they had been objectified and moved around like a piece of furniture. They constantly had to move to new facilities, such as the operating theatre, the ICU, the step-down unit, regular wards, the X-ray department, cramped cubicles, or rooms in the basement. Sometimes they did not even know where they were. This also meant that they changed fellow patients several times. When recovery from surgery took longer than anticipated, they were transferred to their local hospital. The journey between hospitals by bus, by ambulance or by plane could last several hours. Being frail and exhausted after the surgery could mean that the transport was experienced as an unbearable ordeal. However, some participants did not experience room changes or transport as bothersome.

“…the worst part for me was being transported home. It was really difficult. I had a nurse beside me the whole time, but it was a bumpy road, and that is something I don’t wish on any other heart patient to ever have to experience. Throughout my ordeal, I never felt worse than just then”. (female, 80)

### Feeling safe

Despite the unpleasant experiences of cardiac surgery, all participants mentioned that they felt safe and well looked after. Participants expressed their deep gratitude towards health care professionals. Recovering from surgery became a pleasant experience despite all troubles during the hospitalization. They had received excellent care. Also, they expressed gratitude for getting cardiac surgery and being given a chance at renewed life. They felt safe when professionals were competent and helpful and saw them as a person. The sub-themes related to the theme Feeling safe were: “being in supportive hands” and “feeling grateful”.

#### Being in supportive hands

Participants related how they were not worried before surgery because they were convinced that they would receive the best possible help and care.

“…my brother had an operation before I did, and everything went fine for him. So I thought I’m sure I’ll be fine, too”. (male, 87)

Preoperative information, and especially the first meeting with the surgeon, was described as important despite participants finding the information difficult to understand. Preoperative information made participants confident even though it informed them about possible complications. One participant described how surprised he was to feel confident and safe as he was usually an anxious person. However, participants firmly trusted the surgeon’s decision when being promised that surgery would go well.

Relatives and friends visiting during the hospitalization were described as crucial support. Participants described feeling secure and safe, knowing that relatives and friends were nearby, watching over them. When support from relatives and friends did not materialize, participants turned to their fellow patients instead. Fellow patients gave them friendship and support and made the hospitalization bearable.

“Nice people lying there. So we just lay there and talked about everything. How things had gone and so on”. (male, 81)

The participants also described support from physiotherapists as a positive experience. Physiotherapists made them feel confident to exercise and regain control of their body. Participants described the hospital as a luxury hotel with a welcoming atmosphere. Health care professionals were easy-going, cheerful and friendly and had a positive attitude. Participants were prepared for what was going to happen, and received the help they needed. The health care professionals also took the time to sit with, ask questions and talk to them, like new friends.

“I felt sure they would take good care of the patients, and so on. There were no problems. You got whatever you needed. The nurses there were extremely nice”. (male, 79)

#### Feeling grateful

Participants described it as amazing and unbelievable to have the opportunity to undergo cardiac surgery. They were extremely grateful to the health care professionals for positive and good experiences during the hospitalization. Some participants described cardiac surgery as a piece of cake, as everything had gone smoothly and the days in hospital had passed quickly. Hence, they had no concerns about their bodies or their experiences of a weird world.

“…but I was so darn alert! I had my operation during the day or in the morning. Was it the morning after? I walked down to the cafeteria using a walker”. (male, 85)

Participants who experienced cardiac surgery as difficult, demanding and severe were confident in their recovery and grateful for the care and treatments received despite all concerns. They were overwhelmed and grateful for the chance to have woken up after surgery. They had tears of joy when given to understand that they could now live for several more years. It was described as a relief and a rediscovered freedom to survive the cardiac surgery.

“I only feel well, joy and gratefulness, for still existing that I can be with my family”. (male, 74)

## Discussion

This study aimed to illuminate experiences of undergoing cardiac surgery among older people diagnosed with postoperative delirium, a one year follow-up. The findings show that cardiac surgery is a complex, multifaceted and distressing procedure for older people and could present unexpected challenges and suffering. Participants described how they were extremely vulnerable around the time of surgery, and how the surgery became frightening, traumatic and unpleasant. On the other hand, for some, pleasant or rewarding aspects were added to the experience. Feeling drained of viability, feeling trapped in a weird world and being met with disrespect were some of the traumatic and unpleasant experiences. Experiences of good care included feeling safe, being in supportive hands and feeling grateful were also expressed. Participants were grateful for receiving the gift of a second chance in life. It appeared that they had felt safe and secure and were satisfied with the care after all, even those who had experienced discomfort and suffering.

This study indicates that cardiac surgery is a distressing procedure, and patients undergoing cardiac surgery do not take the future for granted. They know they are fragile and close to death, which has been shown elsewhere in similar studies [28,29]. Gardner et al. [7] report that patients’ memories and experiences of cardiothoracic surgery can be demanding and difficult. Going through the procedure of surgery made participants lose the spark of life. Participants in the present study were similarly vulnerable and frail, which was interpreted as feeling drained of viability, as participants felt totally in the hands of health care professionals and had to adapt to the care that was provided. It further appears that participants’ self-image was damaged: they were no longer the person they wished to be. Their pride had become violated when they felt they were a burden to the health care professionals. In the present study delirium made participants lose their self-control and dignity and this made them feel embarrassed. This is in line with findings from previous studies in other setting like orthopedic care, ICU care and major vascular surgery care [30-33]. Stenwall et al. [34] showed that when patients did not trust the staff, who wanted to help them and wanted to understand their experiences of confusion, they avoided exposing their confusion to protect themselves which also is in line with findings in the present study. Some participants had only fragmented memories of cardiac surgery. As a consequence, they felt out of control and had to ask what happened during the surgery. Not knowing what actually happened to you could be seen as a form of suffering from care. Patients suffer when they do not understand what's going on and are unable to decide about their daily life and care [35].

Participants clearly remembered their delirium with unresolved feelings of anxiety, which affected them even one year after cardiac surgery. They described in detail how they were “trapped” in a weird world, out of control. Patel et al. [36] showed that decreasing noises in the care environment can prevent development in delirium. As early as 1965, Kornfeld et al. [37] interviewed patients after going through cardiac surgery. Patients described the frightening atmosphere of postoperative care and the sense of being changed. They did not report their experiences to the staff, and not until more dramatic things happened did they become paranoid and agitated [37]. Similar findings have been described by Laitinen [32] after cardiac surgery and by others in other settings [30,31,38]. An important question to raise is why, despite what is known about experiences of postoperative delirium, so little seems to have been achieved in improving care for these patients.

Delirium is a distressful experience, both for those who remember and for those who do not remember their episodes of delirium [39]. Some participants in the present study said that they did not remember, or barely remembered their experiences of delirium: This is consistent with other studies [30,31]. But, patients may not have the courage to tell about their experiences cf [30] and this may have been the case in this study. Health care professionals may also deliberately avoid asking patients if they can remember being delirious, as they might want to spare them further distress or because they might assume they do not remember.

The present study shows that it is important to address the issue of delirium as a distressful experience. All participants were diagnosed with postoperative delirium, but some were convinced they had not been delirious. Still they described, in detail, their experiences of nightmares and delusions. This indicates that the meaning of the concept “delirium” could differ for different patients, and may not always be consistent with health care professionals’ understanding of the concept. It seems that nurses and doctors should ask patients about unusual experiences, such as nightmares and delusions, instead of experiences of delirium. Participants in the present study were also exposed to fellow patients’ experiences of delirium and hence may have needed explanations and support from nurses. Laitinen, 1996 puts it as follows: “others in the environment may also fall victim to its chaos” ([32], p 82). Stenwall et al. [34] found that patients search for answers to what happened, and why it happened, and they are greatly in need of reflecting on and understanding what happened during their confusion. Another study showed that patients in medical or surgical wards were worried that nurses thought they were complaining and troublesome, and hence endured a lot of suffering before asking for help. Only when the discomfort of their suffering became bigger than the suffering itself, they finally asked for help [40]. In order to make patients feel respected and safe, nurses should be present and open to patients’ situations. Only then they might feel comfortable to ask any questions they might have, related to the hospitalization.

Part of feeling that they were met with disrespect was due to being moved many times during the hospitalization: “feeling like cargo”. The hospital environment was perceived as poor and disturbing, with patients being forced to adapt to health care routines and procedures, without any choice. The cardiac surgery department is designed for constant relocating between various levels of care. This means great challenges for the nurses, who still need to recognize and understand patients’ needs, to provide continuous care and support. It has been argued that a poorly designed physical environment makes staff effectiveness decrease as attention is taken away from patients and patient safety [41]. Today, hospitalization in general has become shorter, but the care provided must still be based on individual needs. Older patients need security, peace, tranquility and continuity. They need to be recognized as people with special needs both physically and mentally, and to be supported and comforted. It should not be as one participant described, that he felt stupid when asking for an explanation for his weird experiences and was met with disrespect. Older patients require more, not fewer, bedside skills for prevention, timely detection, recognition and monitoring of delirium [42,43]. Counteracting experiences of being met with disrespect, as reported in this study, can be possible.

It should be noted that all participants described feeling safe during cardiac surgery despite their difficult experiences. Participants felt safe when staff was competent, helpful, friendly and attentive. Studies have shown that patients have trust in health care and show a lot of confidence in staff after cardiac surgery [28,44]. There are different dimensions of the quality of care, such as being helped at the right time or receiving a bit of kindness. In the present study, participants described health care professionals as new friends when they took time to sit down and talk to them. Laitinen [32] described the importance of human closeness, such as having a professional person treat them, whom the patient knows and does not feel alienated from. In Laitinen’s study, some patients told about experiencing true presence in nursing, manifested in the nurses’ way of speaking, touching, gesturing – even in silent presence [32].

The significance of being heard and seen has also been described in other contexts [45,46]. According to Melnechenko [47], you need to be present in order to see a person. This presence “does not require more time, rather it is as a willingness to focus on really being there and being involved when with another” [47]. Presence of family and comradeship with other patients are important aspects of feeling safe during hospitalization. Sharing experiences with someone who has been through a similar event may be more fruitful than sharing with health care professionals [5,48]. Stenwall et al. [34] showed that patients with delirium felt safe when health care professionals and relatives understood, supported and trusted them during their episode of confusion. In the present study feeling safe included feeling grateful, e.g. for receiving the gift of a second chance in life. This is in line with Karlsson et al. [28] who reported how feelings of joy and relief took over when the surgery was over.

Our findings suggest that it is of great importance to draw the whole picture of older patients’ experiences of cardiac surgery. The present study indicates that health care professionals do not always have the understanding, knowledge, and/or the time and ability to explain to and support patients, and this may further lead to unnecessary suffering. Patients need all their strength to recover and should not have to waste their energy on delirium or distress. Providing support can therefore lead to patients having better ability to fend for themselves.

### Implications for practice and research

This study indicates that health care professionals need to gain understanding of older peoples’ experiences of cardiac surgery, as well as experiences of delirium, to reduce unnecessary suffering. Patients as well as relatives should be informed about and become aware of the occurrence of unfamiliar experiences due to cardiac surgery. Easy to understand information on procedures and routines and on the risk for developing delirium should be provided to make patients and their relatives feel safer. As relatives often play an important role in the process of making patients feel safe they should be included in caring for and supporting patients. Furthermore, nurses are responsible for nursing care, and as those at the bedside need to support patients, and must prevent, detect and treat complications such as delirium. Nurses should venture to ask, even if this is awkward, questions about patients´ experiences of delirium. By being persistent asking questions and, most important, listening to patients and their relatives, nurses can gain understanding of their experiences. Patients should also be offered follow up contacts post-hospital to help them understand what has happened and offered counselling if appropriate.

Delirium is a multifactorial syndrome with severe consequences for patients. Therefore all health care professionals need increased knowledge on how to prevent and treat postoperative delirium in high-risk patients in order to provide good nursing care. It has been recommended that interdisciplinary teams, formal education, various interventions e.g. non pharmacological interventions, environmental adjustments and specialized units could provide better options for care of older people at risk for delirium [49]. Solid research on effective preventions as well as interventions is needed to improve nursing care for older people undergoing cardiac surgery.

### Study strengths and limitations

The strengths of this study are, among others, the large number of participants, which provides a wide range of experiences of cardiac surgery. Participants already knew the two interviewers as they had been involved in patient care during the hospitalization. This could be seen as a limitation as participants might have tried to give the ‘right’ answers. We consider it to be a strength because it may have created more trustful interview situations and hence richer interviews as participants shared painful as well as positive experiences. In order to enhance trustworthiness, all authors were involved in the analysis process. The interviewers were not experienced interviewers. Their pre-understanding as registered nurses responsible for participants during their hospitalization was discussed in the research group throughout the analysis process to clarify its significance for the research process. We also reflected on our findings in relation to relevant literature. An independent research group experienced in using qualitative content analysis evaluated the reasonableness of the results. A text never has one single meaning and there is always some degree of interpretation when approaching a text [25,50]. Our interpretation should therefore be considered one of many plausible understandings.

## Conclusions

This study has provided an understanding of participants’ experiences of undergoing cardiac surgery as described one year post-surgery by older participants diagnosed with postoperative delirium. The participants had felt dependent and totally in the hands of health care professionals. They still had painful memories of their surgery and hospitalization, memories that caused them concern. Despite these memories, the participants also remembered feeling safe and being satisfied with the care provided. To reduce unnecessary suffering, more attention should be given to educating staff, preparing patients and their relatives, and developing procedures for preventing, detecting and treating delirium. It seems reasonable to believe that conclusions from this study could be transferable to other contexts where older people are exposed to complicated treatments or undergo surgery.

## Abbreviations

CPB: Cardiopulmonary bypass
DSM-IV TR: Diagnostic and Statistical Manual of Mental Disorders. IV, Text Revision
ICU: Intensive care unit
